# Analysis of the Innate Immune Response to Febrile UTI in Infants: Evidence of an Acute Cytokine Storm

**DOI:** 10.1097/INF.0000000000004914

**Published:** 2025-08-26

**Authors:** Shahram Ahmadi, Therese Rosenblad, Samudra Sabari, Magnus Linden, Per Brandström, Sing Ming Chao, Catharina Svanborg, Ines Ambite

**Affiliations:** From the * Division of Microbiology, Immunology and Glycobiology, Department of Laboratory Medicine, Lund University; † Department of Pediatrics, Lund Children’s Hospital, Lund; ‡ Department of Pediatrics, Halland Hospital, Halmstad; § Department of Pediatrics, Institute of Clinical Sciences, Sahlgrenska Academy, University of Gothenburg; ¶ Pediatric Uro-Nephrology Center, Queen Silvia’s Children’s Hospital, Gothenburg, Sweden; ∥ Department of Pediatrics, Nephrology Service, KK Women’s and Children’s Hospital, Singapore, Singapore.

**Keywords:** febrile urinary tract infection, cytokine storm, urine proteomics, gene expression, immune response, COVID-19

## Abstract

**Background:**

Infections trigger complex immune responses, with conserved as well as disease-specific characteristics.

**Methods:**

Proteomic screening technology and gene expression analysis were used here to define the immune response in infants, with their first episode of febrile urinary tract infection. Urine and peripheral blood samples were obtained at enrollment and at follow-up, after 6 months.

**Results:**

Pair-wise proteomic analysis of urine samples detected a broad local cytokine response in urine; 20 of the 24 proteins were strongly activated during acute infection compared with follow-up. Functional profiling identified the response as a cytokine storm, and major innate immune response regulators and effector molecules were activated, such as IL-1α, IL-1β, IL-1RA, IL-33, IL-8, IP-10, MCP-1, MIP-1α, MIP-1β, GM-CSF, IL-6, IL-17, TNF-α and IFN-γ. In addition, the adaptive immune response was activated, including CD40L, IL-2, Granzyme B, IL-10, IL-15 and PD-L1. Gene expression analysis of peripheral blood RNA detected a systemic cytokine storm profile, which was more pronounced in infants with renal involvement, defined by positive acute dimercaptosuccinic acid scans, than in infants with febrile urinary tract infection without renal involvement.

**Conclusions:**

Local and systemic hyperactivation of innate immunity characterizes acute pyelonephritis, a common and severe bacterial infection in childhood and a significant cause of urosepsis and mortality in adults. The results define a transient cytokine storm response, resembling that induced during severe acute respiratory syndrome coronavirus 2 infection, as characteristic of acute pyelonephritis, rather than individual protein biomarkers.

The innate immune response maintains the first line of defense against infections, and when efficient, innate immunity stops pathogen attack and restores health.^1^ In contrast, a loss of immune control may create severe disease as the immune response loses its protective powers, and innate immune hyperactivation has been identified as a cause of disease in susceptible individuals.^2,3^ Understanding the molecular basis of innate immune dysregulation is therefore essential, not least to discover new approaches for targeting and therapeutically correcting dysfunctional immune responses in susceptible individuals.^3–5^

Febrile urinary tract infection (fUTI) is 1 of the most common bacterial infections in infancy and childhood and a significant concern, both diagnostically and therapeutically.^6,7^ The clinical presentation of fUTI in infants includes signs of systemic illness such as fever, irritability, poor feeding and lethargy.^8^ Early studies in murine UTI models analyzed, in great detail, how the innate immune response controls the antibacterial defense and severity of UTI. Clinical studies further identified cytokines such as IL-6 and IL-8 as activated in this patient group.^9–12^ While systemic host response parameters like leucocyte counts and C-reactive protein (CRP) levels are widely used diagnostically and leukocyturia is indicative of UTI,^13–15^ there are at present no innate immune response parameters that can accurately grade acute disease severity or identify the subset of patients, who develop acute pyelonephritis or renal scarring.^16^

Technologic advances now make it possible to investigate the extent of immune activation and the profile of regulated cytokines and immune response regulators. A recent study showed that infants with acute pyelonephritis are genetically distinct from the group with fUTI without renal involvement, using DNA exome sequencing. Gene expression analysis and proteomic tools further characterized the molecular disease response, suggesting that innate immune hyperactivation is a characteristic of fUTI, and specifically of acute pyelonephritis.^17^ In this study, innate immune activation was analyzed in a second population of infants with their first fUTI episode. Proteomic screening technology was used to quantify the local immune response in the urinary tract and gene expression analysis to investigate the systemic response. A local response, characterized as a cytokine storm, was identified in acute samples, consistent with the clinical presentation, and severity of acute disease and the systemic involvement, which characterizes fUTI, was confirmed by gene expression analysis.

## METHODS

### Study Populations

The samples were obtained from a subset of infants included in a prospective multicenter study of infant UTI conducted at 29 pediatric centers in Sweden during a period of 3 years. The study was approved by “Regionala etikprövningsnämnden Lund” according to protocol diary number 2015/884. Complementary ethical applications were approved (diary number 2016/799, 2017/164 and 2017/315). Written and oral information was presented to the children’s caretakers, and participation required written informed consent. Detailed analysis of the immune response in infants diagnosed with a first episode of fUTI was conducted at 3 of the centers according to Swedish guidelines. Urine samples and RNA samples were collected within 24 hours of diagnosis and at the scheduled follow-up visit after 6 months.

Children less than 1 year of age were recruited from pediatric emergency units when fUTI was suspected. Diagnostic criteria were a positive urine culture with growth of a single uropathogen of ≥10,000 colony-forming units (cfu)/mL, temperature >38.0 °C, pyuria and CRP ≥20 mg/L after a fever duration of at least 24 hours. Exclusion criteria were a past history of UTI and major renal or urologic abnormalities. Blood samples were obtained for CRP and creatinine levels, and urine sample for dipstick and culture. Infants with confirmed fUTI were investigated by dimercaptosuccinic acid (DMSA) scan within 7 days from the start of antibiotic treatment. Infants with 1 or more focal uptake defects (DMSA+) were diagnosed as acute pyelonephritis and the others as fUTI without renal involvement (DMSA−). In patients with a first positive DMSA scan, a follow-up DMSA scan was performed after at least 6 months to detect renal scarring.

### Proteomic Analysis of the Response to Infection in Urine Samples

Urine was stored at −20 °C until transport within 1 hour of sampling and transferred to a −80 °C freezer. Frozen samples were transported to the Department of Microbiology, Immunology and Glycobiology and further stored at −80 °C. Urine samples were obtained at enrollment from 55 patients with fUTI. Urine samples were further obtained at follow-up after 6 months from 29 patients. Renal involvement was detected by a positive first DMSA scan in 33/55 patients with acute samples. Paired samples were obtained from 20 patients with first DMSA+ and 9 patients with first DMSA−.

Urine samples were analyzed with the Luminex-based assay. The concentration of 24 cytokines and chemokines was analyzed. The Human Immunotherapy Luminex Performance Assay 24-plex Fixed Panel was used to quantify CCL2/MCP-1, CCL3/MIP-1α, CCL4/MIP-1β, CD40Ligand/TNFSF5, CXCL-10/IP-10, GM-CSF, Granzyme B, IFN-α, IFN-γ, IL-1α, IL-1β, IL-1RA, IL-2, IL-4, IL-6, IL-8/CXCL8, IL-10, IL-12 p70, IL-13, IL-15, IL-17/IL-17A, IL-33, PD-L1/B7-H1 and TNF-α (R&D Systems, Minneapolis, MN, Cat #: HCYTMAG-60K-PX41, Lot #: 3090739). All assays were validated by the manufacturer for sensitivity, intraassay precision and assay linearity. Assays were tested for less than 0.5% cross-reactivity and interference. After thawing on ice, the urine samples were gently mixed and used undiluted. Reverse pipetting was used for high accuracy in all liquid handling steps. High and low controls, all standards and buffer controls were analyzed in duplicate. The assays were performed according to the manufacturer’s instructions. In this protocol, standard, low and high controls were reconstituted with calibrator diluent, allowed to sit for 15 minutes, followed by 5-minute gentle agitation. Standard 1 was used for 3-fold dilution series using diluent calibrator diluent. Briefly, 50 μL of different standard (7-highest to lowest), controls (low and high) or sample were added to each well and the 50 μL diluted microparticle cocktail (0.5 mL microparticle cocktail + 5 mL microparticle diluent) was added, subsequent foil plate sealing and 2-hour incubation at room temperature on a microplate shaker. Washing involved applying a magnetic device, filling wells with wash buffer (100 μL) and repeating this process 3 times. About 50 μL of diluted biotin-antibody cocktail (0.5 mL biotin-antibody cocktail + biotin-antibody diluent 2) was added, followed by a 1-hour incubation and a repeat of the wash steps. Subsequently, 50 μL of diluted streptavidin-phycoerythrin (220 μL streptavidin-phycoerythrin—concentrated + 5.35 mL wash buffer) was added, and after a 30-minute incubation, a final round of washing was performed. Microparticles were resuspended with wash buffer (100 μL per well) and incubated for 2 minutes on the shaker.

Reading was carried out within 90 minutes using a Luminex (Austin, TX) or Bio-Rad (Hercules, CA) analyzer, emphasizing immediate microparticle resuspension before reading by shaking the plate for 2 minutes at 800 ± 50 rpm. The fluorescence intensities of a minimum of 100 beads/analytes were recorded using the Bio-Plex Manager 6.0 Software (Bio-Rad) calibrated according to the manufacturer’s instructions. The median fluorescence intensity was used for standard curve fitting and quantitation of cytokine concentrations. Standard curves were fitted using 5-parameter logistic regression with 4-parameter logistic regression as a fallback for occasions where the 5-parameter logistic regression model would not converge. The concentrations of 2 technical replicates of each standard and controls were averaged before statistical analysis. Concentration values were calculated and analyzed using the GraphPad Prism (v.10.0.2, Boston, MA) program, based on the standard curves.

### Gene Expression Analysis

RNA was stabilized and purified from peripheral blood collected at the time of diagnosis and 6 months postinfection using Tempus blood RNA tubes and purification kit (Applied Biosystems, Waltham, MA). RNA was subjected to expression microarray analysis: 100 ng of total RNA was amplified using the Affymetrix WT PLUS Reagent Kit and hybridized using the GeneTitan system onto GeneChip Human Gene 2.1 ST Arrays (all from Affymetrix, Santa Clara, CA) with probe sets measuring the expression of 72,688 transcripts, including a large number of noncoding and hypothetical transcripts. Follow-up RNA samples were used as controls.

Transcriptomic data were normalized using the Robust Multi-chip Analysis algorithm implemented in the Transcriptome Analysis Console software (v.4.0.1.36, Applied Biosystems, Thermo Fisher Scientific, Waltham, MA). The Transcriptome Analysis Console software calls the limma differential expression portion of the Bioconductor package to provide fold change (FC). FCs were calculated by comparing acute samples with follow-up samples obtained 6 months after enrollment. Relative expression was analyzed by analysis of variance using the empirical Bayes method and Benjamini-Hochberg step-up false discovery rate-controlling procedure at an alpha of 0.05 to correct for multiple comparisons. Genes with a *P* value <0.05, a false discovery rate-adjusted *P* value <0.05 and an absolute FC >1.5 were considered differentially expressed. Differentially expressed genes and regulated pathways were analyzed using Ingenuity Pathway Analysis software (IPA, Qiagen, Venlo, Netherlands), using right-tailed Fisher exact test followed by Benjamini-Hochberg correction for multiple testing.

### Statistical Analysis

*P* values <0.05 were considered significant. For proteomics, as all data were not following a Gaussian distribution (D’Agostino and Pearson normality test), nonparametric analysis tools were used. Pair-wise analysis of urine proteomic data was performed using Wilcoxon signed-rank test, comparing protein concentrations at the time of acute UTI with follow-up. For group-wise analysis, Mann-Whitney *U* test was used to compare protein concentrations in urine samples. For FC calculations of proteomics data, log_10_ concentration values were compared between acute and follow-up samples, followed by statistical analysis using Student *t* test. Gene expression was tested using using right-tailed Fisher exact test followed by Benjamini-Hochberg correction for multiple testing. Statistical significance was determined using Microsoft Excel or GraphPad Prism (v.10.0.2).

## RESULTS

Infants with suspected fUTI were included in this study (23 boys; 32 girls; mean age, 4.2 months) (Figure, Supplemental Digital Content 1, https://links.lww.com/INF/G315). A diagnosis of fUTI was confirmed by a positive urine culture (single growth >10^4^ cfu/mL in catheterized urine or >10^5^ cfu/mL in midstream clean-catch urine), a temperature >38.5 °C, pyuria and increased CRP levels. Acute pyelonephritis was diagnosed in 33/55 infants by a positive first DMSA scan within 7 days of inclusion (mean CRP level, 96.9 mg/L). The remaining 22 patients were diagnosed with fUTI without acute renal involvement (mean CRP level, 69.3 mg/L). Dilating vesico-ureteric reflux grade III–IV was detected in 6/27 children investigated by micturating cysto-urethrogram and 2 had nondilating vesico-ureteric reflux (grade I–II) (Figure, Supplemental Digital Content 1, https://links.lww.com/INF/G315). Two patients were subjected to surgery because of obstructive malformations (uretheric cele and meatus stenosis). The predominant pathogen in urine cultures was *Escherichia* *coli* (50/55).

### Proteomic Analysis of the Host Response at Recruitment Versus Follow-up

Urine samples obtained at enrollment were subjected to Luminex protein panel analysis and compared with samples obtained from the same population at follow-up, after about 6 months (n = 29 patients, 58 urine samples). An additional 26 urine samples were obtained at enrollment without follow-up (n = 26). The concentration of each cytokine was quantified relative to a standard curve (pg/mL of urine).

**FIGURE 1.**
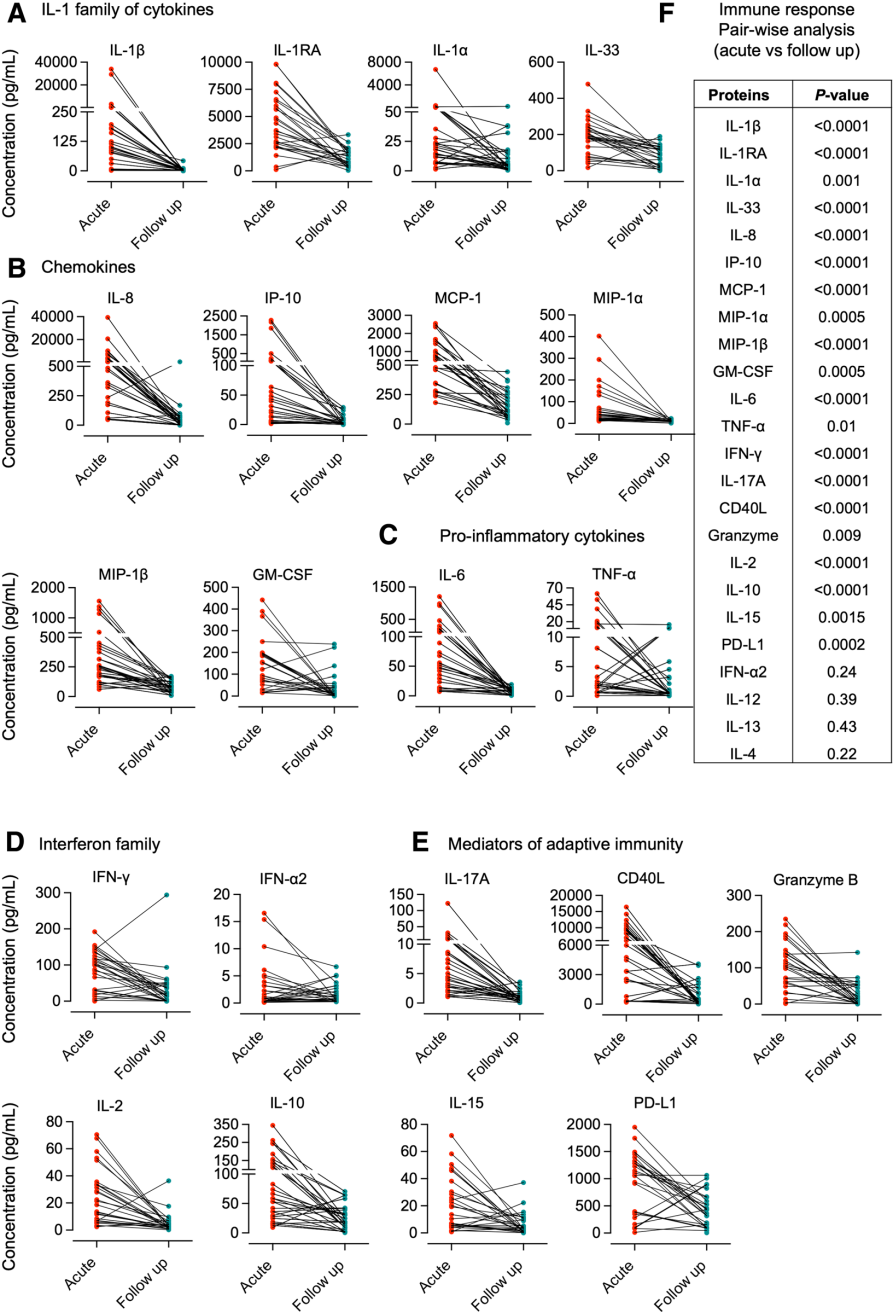
Pair-wise analysis of the cytokine response to fUTI at enrollment compared with follow-up.A–E: Elevated cytokine concentrations in urine at the time of enrollment compared with follow-up in individual patients. Activated cytokines included IL-1β, IL-1RA, IL-1α, IL-33, IL-8, MCP-1, IP-10, MIP-α, MIP-β, GM-CSF, IL-6, IL-17A, IFN-γ, TNF-α, CD40L, PD-L1, Granzyme B, IL-10 and IL-15. The cytokines IFN-α2, IL-12, IL-13 and IL-4 were not significantly regulated. F: Statistical analysis of cytokines in urine at the time of infection compared with follow-up (*P* values, Wilcoxon signed-rank test).

A potent cytokine response to acute infection was detected by pair-wise analysis, comparing the acute with the follow-up samples. The majority of cytokines (20/24 cytokines) showed significantly elevated levels at enrollment. A significant increase in cytokine concentration was detected for the IL-1 family of cytokines, including IL-1β, IL-1RA, IL-1α, IL-33 (Fig. 1A), chemokines and proinflammatory cytokines (IL-8, IP-10, MCP-1, MIP-1α, MIP-1β, GM-CSF) (Fig. 1B), IL-6, TNF-α, IFN-γ (Fig. 1C,D) and mediators of adaptive immunity (IL-17, CD40L, Granzyme B, IL-2, IL-10, IL-15 and PD-L) (Fig. 1E) compared with the follow-up samples. In contrast, there was no significant difference in the concentration of IFN-α2, IL-12, IL-13 or IL-4 between acute and follow-up samples (Fig. 1F). The results identify a potent and broad local innate immune response to fUTI.

The median IL-1β concentration at enrollment was 161 pg/mL in the fUTI group, compared with 2.6 pg/mL at follow-up, and the concentration of IL-1α was 20 pg/mL, compared with 5.3 pg/mL at follow-up (Figure, Supplemental Digital Content 2A, https://links.lww.com/INF/G315). The IL-8 chemokine concentration was 600 pg/mL, at enrollment compared with 27.8 pg/mL at follow-up. Other strongly activated chemokines included IP-10 (43 pg/mL at enrollment compared with 2.7 pg/mL at follow-up), MCP-1 (670 pg/mL at enrollment compared with 117 pg/mL at follow-up), MIP-1α (32 pg/mL at enrollment compared with 11 pg/mL at follow-up), MIP-1β (258 pg/mL at enrollment compared with 82 pg/mL at follow-up) and GM-CSF (94 pg/mL at enrollment compared with 11 pg/mL at follow-up). IL-6, IL-17 and IFN-γ showed a more than 5-fold increase in urine cytokine concentrations, as did TNF-α IL-17, CD40, IL-2, Granzyme B and IL-10. In contrast, no significant difference was observed for IFN-α2 (0.7 pg/mL at enrollment compared with 0.8 pg/mL at follow-up), IL-13 (6.2 pg/mL at enrollment compared with 4.9 pg/mL at follow-up), IL-12 (8.4 pg/mL at enrollment compared with 1.8 pg/mL at follow-up) or IL-4 (0.08 pg/mL at enrollment compared with 0.08 pg/mL at follow-up) (Figure, Supplemental Digital Content 2A, https://links.lww.com/INF/G315).

The analysis of the acute disease samples was extended to include additional patients, from whom acute samples were obtained (n = 26). The acute samples were pooled for analysis based on a comparison of the acute samples from the 2 groups that revealed a high degree of similarity (Figure, Supplemental Digital Content 2B, https://links.lww.com/INF/G315). No significant differences between the groups was found for 19/24 proteins (IL-1β, IL-1RA, IL-1α, IL-8, IP-10, MCP-1, MIP-1α, MIP-1β, GM-CSF, IL-6, IFN-α, TNF-α, IL-17, IL-2, IL-10, IL-15, PD-L1 and IL-12) (Figure, Supplemental Digital Content 2B, https://links.lww.com/INF/G315).

**FIGURE 2.**
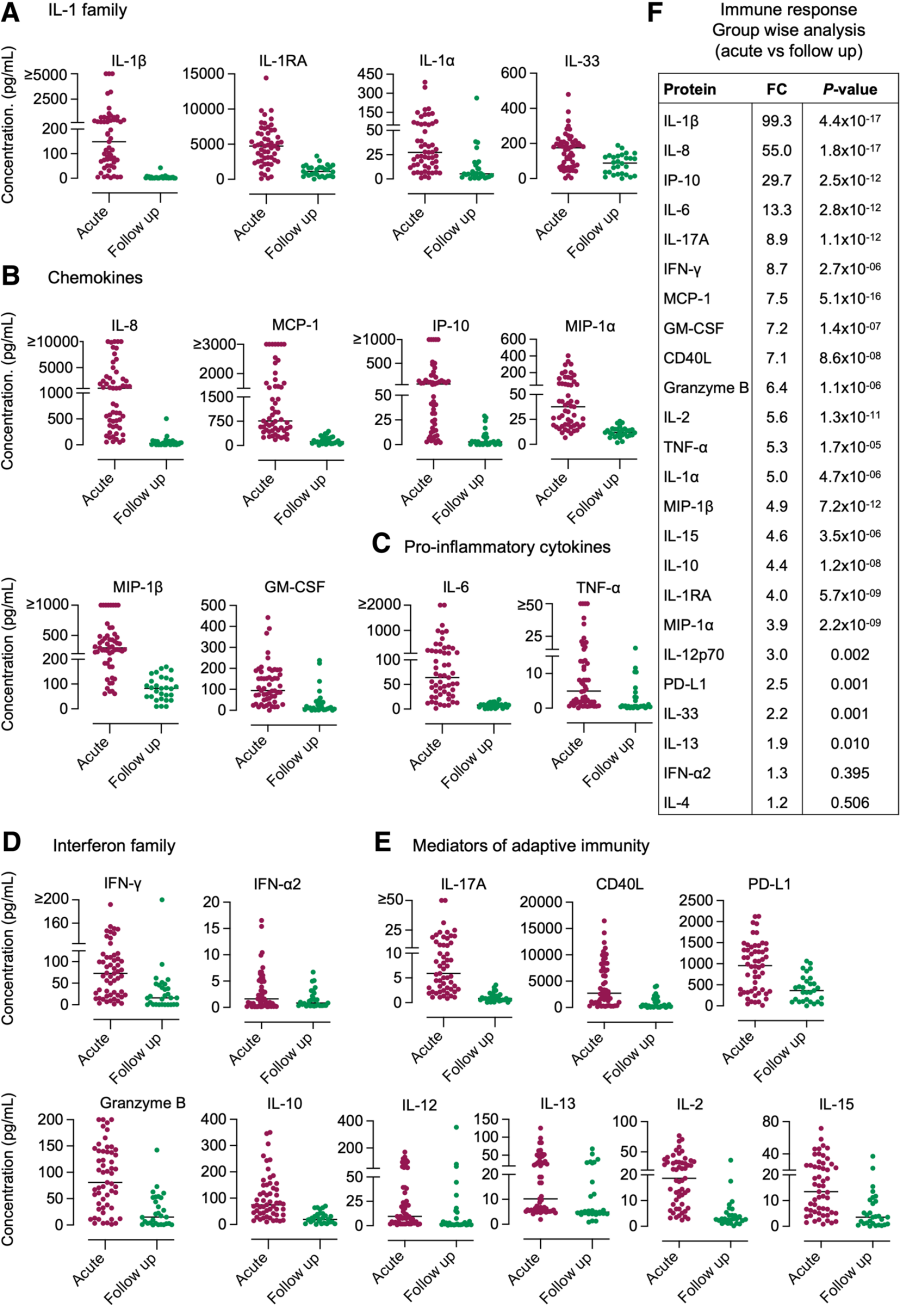
Quantification of the cytokine response in urine samples from infants with fUTI. A: The concentration of the IL-1 family of proteins (IL-1β, IL-1RA, IL-1α and IL-33) was increased at the time of infection compared with follow-up. B: The chemokines IL-8, MCP-1, IP-10, MIP-α, MIP-β and GM-CSF were elevated at the time of infection compared with follow-up. C: The proinflammatory cytokines IL-6 and TNF-α increased in response to infection. D: The IFN-γ response increased significantly during acute infection. E: The adaptive immune cytokines IL-17A, CD40L, PD-L1, Granzyme B, IL-10, IL-12, IL-13, IL-2 and IL-15 showed increased levels in urine at the time of infection, compared with follow-up. F: Statistical analysis of cytokines in urine at the time of infection compared with follow-up (FC = fold change of log_10_ concentration values, *P* values using Student *t* test).

The acute response profile in the pair-wise analysis was subsequently confirmed in the larger group, as activation was detected for 22 of the 24 cytokines analyzed (group-wise analysis, compared with follow-up samples) (Fig. 2). Strongly increased urine concentrations were observed for the IL-1 family of cytokines (IL-1β, IL-1RA, IL-1α and IL-33) (Fig. 2A), chemokines and proinflammatory cytokines (IL-8, IP-10, MCP-1, MIP-1α, MIP-1β, GM-CSF, IL-6 and TNF-α) (Fig. 2B,C), IFN-γ (Fig. 2D) and mediators of adaptive immunity (IL-17, CD40L, IL-2, Granzyme B, IL-10, IL-15, PD-L1, IL-13 and IL-12) (Fig. 2E). The cytokines were further ranked according to the magnitude of the response compared with the follow-up samples (Fig. 2F). IL-1β (FC = 99), IL-8 (FC = 55), IP-10 (FC = 30) and IL-6 (FC = 13) were identified as the most strongly activated cytokines in this extended patient group.

### Cytokine Storm Response

**FIGURE 3.**
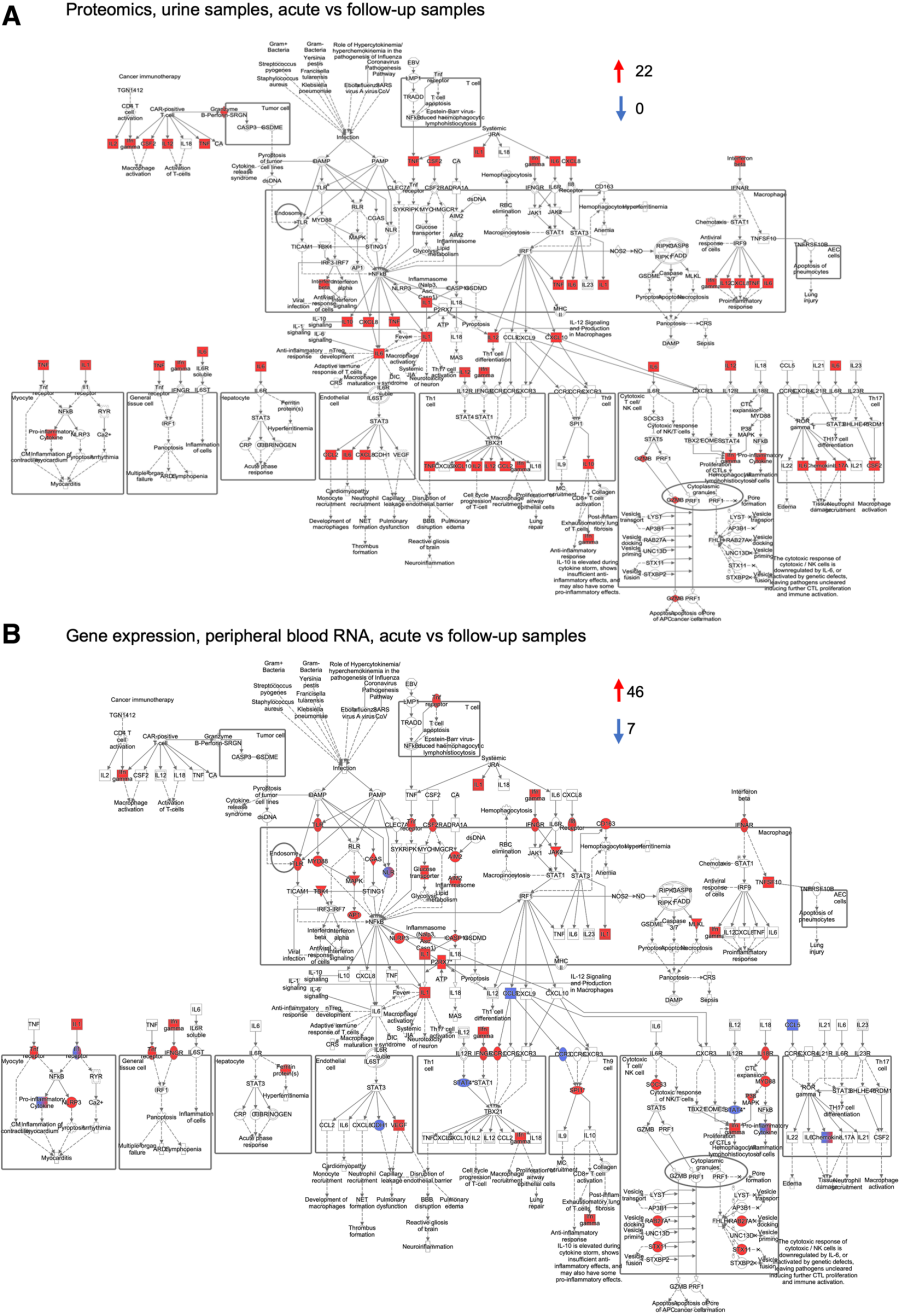
Activation of local and systemic cytokine storm signaling. Significant upregulation of the cytokine storm response in acute urine samples, quantified by proteomic analysis and analyzed by IPA. A: Urine cytokine concentrations were increased in acute urine samples, including IL-1 family of cytokines, proinflammatory and inflammatory cytokines, chemokines and mediators of adaptive immunity in the acute phase of infection compared with follow-up (22 upregulated and 0 downregulated proteins). B: Significant upregulation of cytokine storm signaling pathway genes in peripheral blood RNA, quantified by gene expression profiling of acute versus follow-up samples. Significant activation of innate immunity (46 upregulated and 7 downregulated genes, cutoff FC 1.5 and adjusted *P <* 0.05).

The proteomic profile in the acute urine samples from patients with fUTI (n = 55) was functionally characterized by IPA (Fig. 3A). The cytokine hyperactivation profile was identified by IPA (FC 2, *P <* 0.05) as a cytokine storm, also described in disorders of immune regulation, including severe infections such as COVID-19 and bacterial sepsis.^18,19^

**FIGURE 4.**
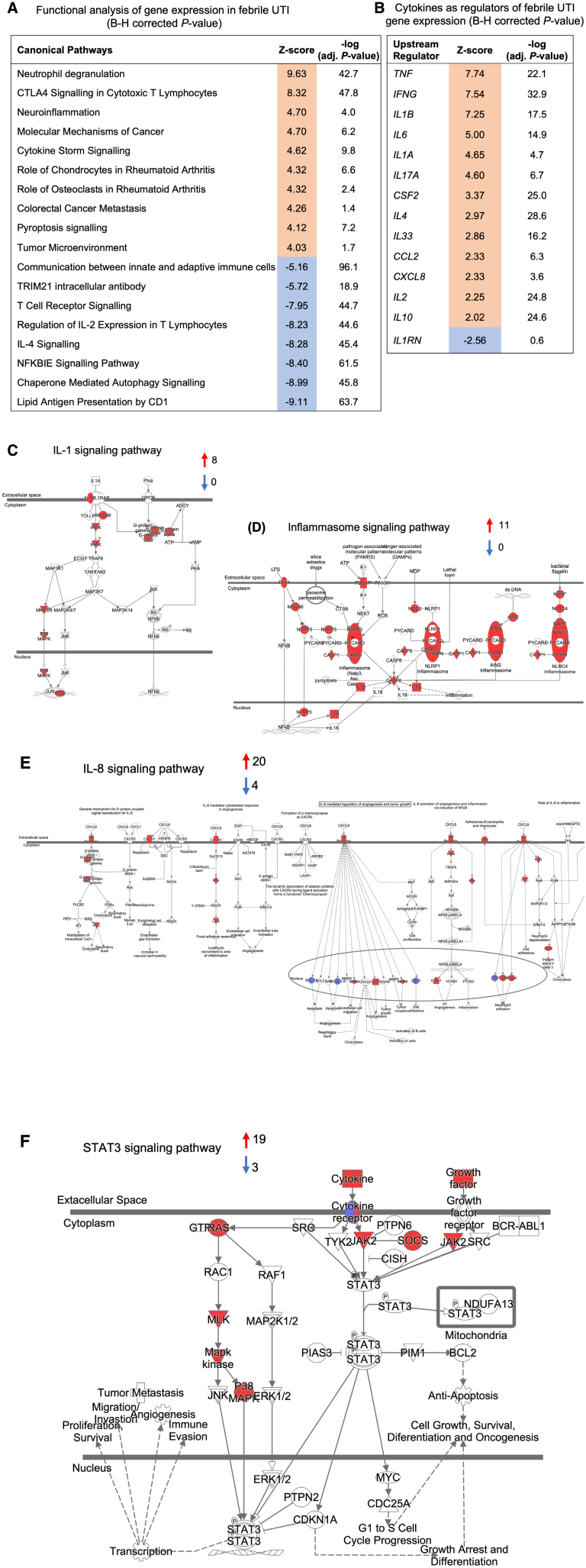
Canonical pathway analysis of genes regulated in patients with fUTI. Regulation of innate immune response pathways, defined by canonical pathway analysis of genes regulated in patients with fUTI (FC 1.5, adjusted *P <* 0.05). A: Significantly regulated pathways at the time of enrollment included neutrophil degranulation, CTLA4 signaling in cytotoxic T lymphocytes, neuroinflammation and cytokine storm signaling. In contrast, chaperone-mediated autophagy signaling, *NFKBIE* signaling, IL-4 and IL-2 signaling were inhibited. *P* values were corrected using the Benjamini-Hochberg procedure (adjusted *P* value). B: Upstream regulators of the systemic gene expression profile included several cytokines predicted to be activated (*TNF*, *IFNG*, *IL1B*, *IL6* and *IL1A*) by IPA. *IL1RN* (gene encoding IL-1RA), as a regulator of the response, was predicted to be inhibited. *P* values were corrected using the Benjamini-Hochberg procedure (adjusted *P* value). C: Activation of genes in the IL-1 signaling pathway in the patients with fUTI at the time of infection, compared with the follow-up samples. D: Activation of genes in the inflammasome signaling pathway in the patients with fUTI, compared with the follow-up samples. E: Strong upregulation of *IL8* and IL-8 receptor genes in patients with fUTI in acute samples compared with follow-up. F: Activation of the STAT3 signaling pathway at the time of infection, compared with follow-up (red = upregulated, blue = downregulated, cutoff FC 1.5, adjusted *P <* 0.05).

The systemic response was analyzed by subjecting peripheral blood RNA to gene expression analysis. Samples were collected at the time of infection from 43 of the 55 patients and at follow-up from 25 patients. The acute response was quantified by comparing acute and follow-up samples. Functional analysis identified a systemic cytokine storm in the entire fUTI patient group (Fig. 3B). The majority of regulated genes were activated (n = 46/53, FC- 1.5, adjusted *P <* 0.05). In addition to cytokine storm signaling, activated canonical pathways identified by IPA also included neutrophil degranulation, pyroptosis signaling, as well as neuroinflammation, IL-8 and IFN-γ signaling pathways, confirming the data from a recent study (Fig. 4A).^17^ In contrast, the expression of genes involved in adaptive immunity was mainly inhibited. The CTLA4 T-cell inhibitor was activated and NF-κB, IL-2, IL-4, T-cell receptor, antigen presentation, TRIM21 and communication between innate and adaptive immune cells signaling pathways were inhibited (Fig. 4A). Key regulators of the systemic response were identified by IPA: *TNF, IFNG, IL1B, IL6, IL1A, IL17A, CSF2, IL4, IL33, CCL2, CXCL8, IL2* and *IL10* were activated but *IL1RN* was inhibited (Fig. 4B). RNA data from 25 of the patients were examined in previously published comparison of Swedish and Singaporean infants with febrile UTI.^17^

Genes in the IL-1 and inflammasome signaling pathways were strongly activated in patients with fUTI at the time of infection, compared with follow-up (Fig. 4C,D). Furthermore, genes in the IL-8 signaling pathway were activated in the entire fUTI group compared with follow-up (Fig. 4E) and genes in the STAT3 signaling pathway were strongly activated (Fig. 4F). Additional activated pathways including IL-6, acute phase response, chemokine and IL-17 signalings were observed at the time of infection compared with follow-up (Figure, Supplemental Digital Content 3A–D, https://links.lww.com/INF/G315). In contrast, NF-κB signaling genes were downregulated at the time of infection compared with follow-up (Figure, Supplemental Digital Content 3E, https://links.lww.com/INF/G315).

The results identify a local and a systemic cytokine response in infants with fUTI, defined functionally as a cytokine storm, involving the overexpression of key regulators of innate immunity and loss of adaptive immune control. The pattern is consistent with the clinical presentation with local and systemic symptoms of infection.

### Cytokine Storm in Febrile UTI Compared With COVID-19

The term cytokine storm has been coined to define excessive, mainly innate immune responses to infection, where multiple response pathways are activated in parallel.^20–22^ Similar complex responses have been identified in patients with severe viral and bacterial infections, suggesting a shared profile of excessive immune activation in patients with severe infections of viral and bacterial origin.^5,20,23^

**FIGURE 5.**
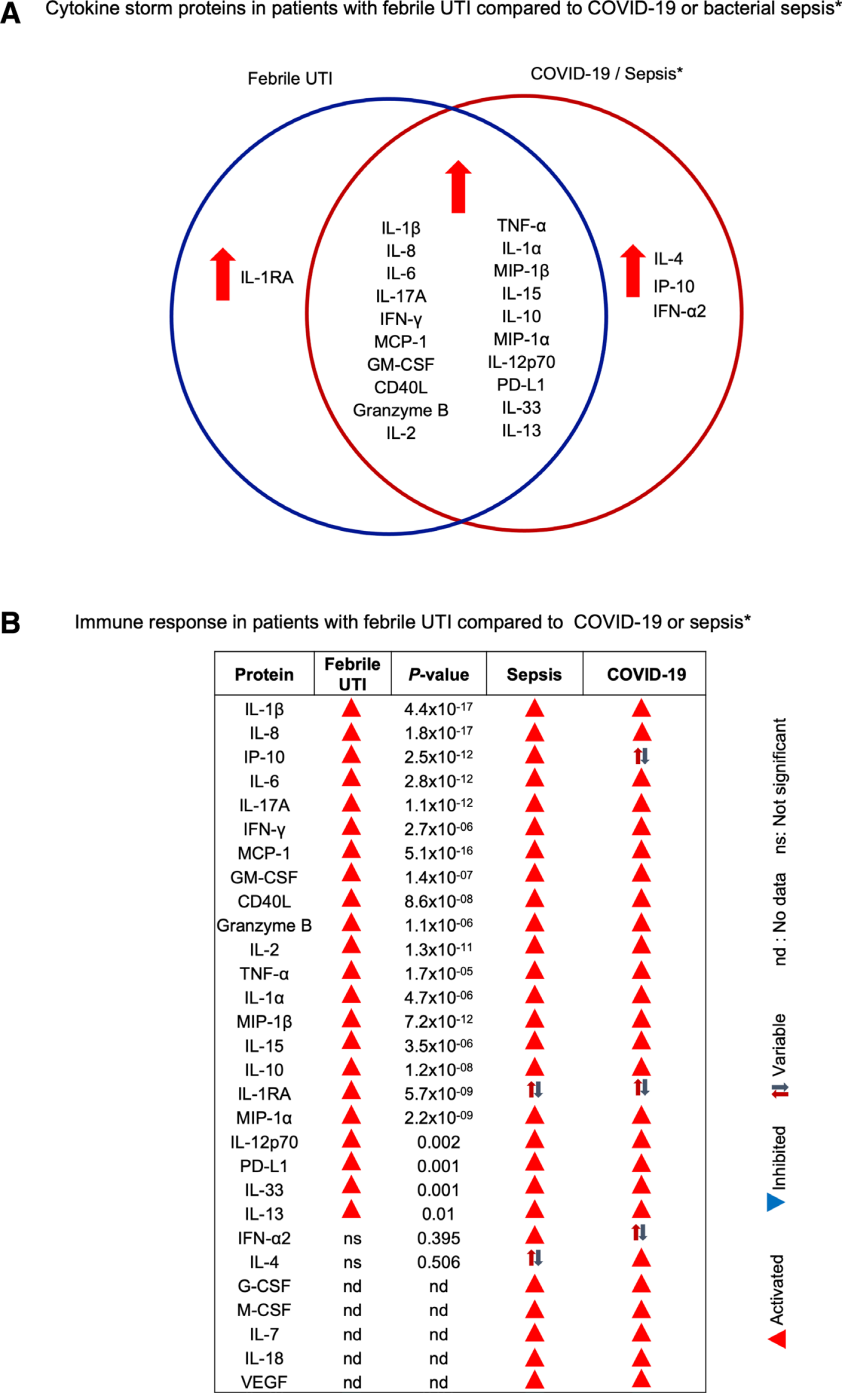
Comparative analysis of the immune response to fUTI and the immune response during COVID-19 or bacterial sepsis. A: Venn diagram visualizing the overlap in cytokine storm profiles between patients with fUTI in this study and COVID-19 or bacterial sepsis, reported in the literature. B: Cytokines regulated in patients with fUTI, severe COVID-19 or bacterial sepsis. Arrows indicate activation (red). *P* values are shown for the immune response to infection described in this study (log_10_ concentration values, *P* values using Student *t* test). nd indicates no data; ns, no significant differences. *Reported in the literature.^5,19–29^

Further analysis revealed a significant overlap in urine cytokine profiles between patients with COVID-19 reported in the literature^21,22,24–26^ and patients with fUTI analyzed in this study (Fig. 5A). Elevated levels of urinary cytokines and chemokines such as IL-1β, IL-8, IL-6, IL-17A, IFN-γ, MCP-1, GM-CSF, CD40L, Granzyme B, IL-2, TNF-α, IL-1α, MIP-1β, IL-15, IL-10, MIP-1α, IL-12, PD-L1, IL-33 and IL-13, detected in this study, have also been reported in patients with severe or critical SARS-CoV-2 infection compared with patients without COVID-19 or with mild or moderate infections (Fig. 5B).^19,27,28^ Additionally, patients with severe or critical COVID-19 showed elevated urinary levels of IL-7, IL-16, IL-18, G-CSF, LIF, CCL-11, CXCL-10, FGF-β, M-CSF, VEGF and CTAcK, which were not measured in this study.^19,20,29^

### Local Proteomic Response and Renal Involvement

**FIGURE 6.**
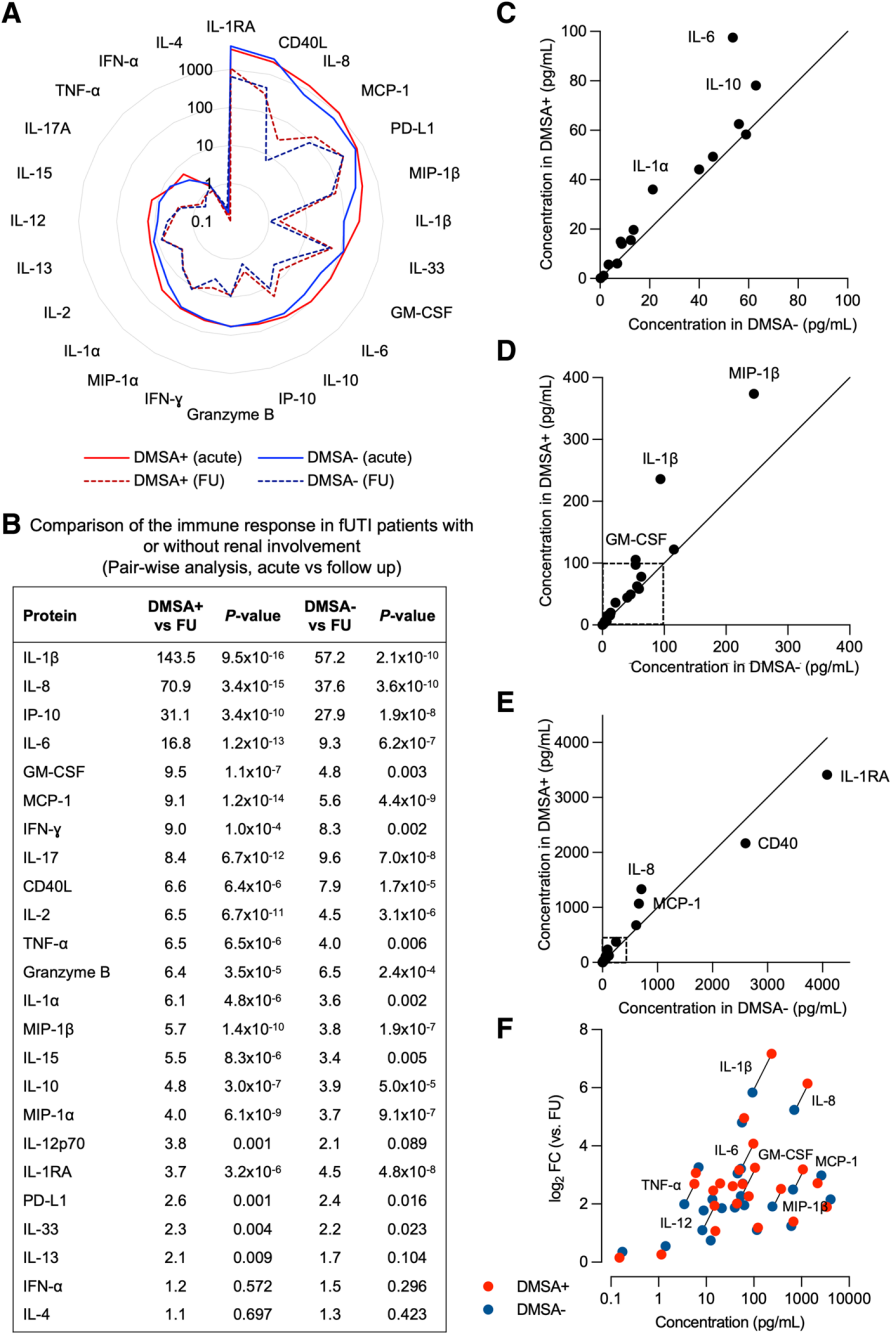
Cytokine responses in patients with fUTI with or without renal involvement (DMSA+ or DMSA−). A: Radar chart comparing mean cytokine levels in patients with or without renal involvement (red line: first DMSA+ acute samples, blue line: first DMSA− acute samples, red dotted line: first DMSA+ follow-up samples, blue dotted line: first DMSA− follow-up samples). B: Acute urine cytokine concentrations in patients with or without renal involvement, compared with follow-up (fold change of log_10_ concentration values, *P* values using Student *t* test). C–E: Direct comparison of mean protein concentrations in patients with or without renal involvement based on different urine concentration ranges (panel C = proteins with low concentration, panel D = proteins with mild concentration, panel E = proteins with high concentration). GM-CSF (*P* = 0.042) and MCP-1 (*P* = 0.046) were significant using Student *t* test for log_10_ concentration values. F: Correlation of urine protein concentrations in patients with or without renal involvement (log_2_ FC), relative to mean protein concentrations.

The cytokine response in urine was further analyzed in relation to renal involvement, by comparing urine samples from the first DMSA+ to the first DMSA− patient groups to the follow-up samples (Fig. 6). The overall analysis is illustrated in the Radar plot in Fig. 6A. The cytokine levels in the DMSA+ patients were consistently higher than that in the DMSA− group. The FC compared with follow-up and *P* values of the regulated cytokines are shown in Fig. 6B, identifying IL-1β, IL-8, IP-10, MCP-1 and IL-6 as the most strongly regulated (Fig. 6B). A direct comparison further identified IL-1α, IL-6, IL-10, IL-1β, GM-CSF, MIP-1β, IL-8 and MCP-1 as more strongly activated in the first DMSA+ than the first DMSA− patient group. In contrast, IL-1RA and CD40L were higher in the first DMSA− than in the first DMSA+ patient group (Fig. 6C–E). The less strongly regulated cytokines showed minor differences between the first DMSA+ and first DMSA− groups (Fig. 6F).

The results suggest that in addition to the overall cytokine storm response pattern, individual cytokines were also more strongly activated in the first DMSA+ than in the first DMSA− group, but the results do not identify single cytokines as specific for fUTI with acute renal involvement.

### Acute Disease Association of the Host Response Parameters

The proteomic analysis was further evaluated using the receiver operating characteristic (ROC) curve analysis, which visualizes the sensitivity and specificity of each protein as a biomarker and the area under the curve (AUC) values, which measure the accuracy of the outcome for parameters used.

The combination of IL-1β and IL-8 was shown to identify patients with acute fUTI, with an AUC value of 0.989 and *P* < 0.0001, compared with concentrations at follow-up. The combination of 3 proteins IL-1β, IL-8 and IL-6 showed an AUC value of 0.990 (*P* < 0.0001) for the ROC curve, indicating a high predictive accuracy (Figure, Supplemental Digital Content 4A, https://links.lww.com/INF/G315). Furthermore, the difference in distribution of the concentrations between acute samples in the DMSA+ or DMSA− group is illustrated in Figure, Supplemental Digital Content 4B, https://links.lww.com/INF/G315. ROC analysis was performed to compare IL-1β, IL-8 and IL-6 concentrations at enrollment between DMSA+ and DMSA− patients as potential biomarkers. The ROC curves indicated that these proteins did not significantly discriminate the DMSA+ from the DMSA− groups (Figure, Supplemental Digital Content 4C, https://links.lww.com/INF/G315).

## DISCUSSION

In this study, infants with their first fUTI episode were shown to develop a potent innate immune response to infection, characterized as a cytokine storm. The term cytokine storm was originally coined to describe the excessive nature of the inflammatory responses to certain infections,^18^ leading to potentially life-threatening immune hyperactivation states, associated with severe viral infections such as SARS-CoV-2 and Ebola as well as bacterial sepsis.^20,23^ Comparative analysis revealed a significant overlap between the fUTI group, studied here, and published analyses of patients with SARS-CoV-2 infection or bacterial sepsis,^21,28,29^ identifying excessive cytokine activation and an out-of-control innate immune response as a cause of disease severity in infants with fUTI. The results confirm and extend recently published data of fUTI in infancy, at the proteomic and gene expression levels.^17^

The molecular basis of acute pyelonephritis has been extensively studied in the murine acute pyelonephritis model and in patients with UTI.^10,30,31^ Uropathogenic *E. coli* activate a TLR-4-dependent innate immune response and single-gene deletions have identified *Tlr4* as an upstream regulator of kidney pathology.^32–34^ Further studies have shown that transcription factors downstream of *Tlr4* regulate disease severity, affecting many of the cytokines identified in this study. *Irf3*^*−/−*^ mice develop severe acute pyelonephritis and renal damage, due to excessive innate immune activation, while *Irf7*^*−/−*^ mice are unresponsive and protected.^10,31^ The *Irf3*^*−/−*^ mice show a susceptibility pattern definable as an “out-of-control” immune hyperactivation state in renal tissues with a cytokine storm profile, excessive neutrophil accumulation, abscess formation and impaired bacterial clearance.^10,31^ Furthermore, the recruitment and activation of neutrophils are essential for the antibacterial defense of the kidney and the IL-1 family of cytokines controls susceptibility to experimental infection in kidneys and bladders.^3,12,17^ The proteomic data and RNA screen described here detected very similar response patterns, extending the molecular observation from animal models to the clinic. The results emphasize the importance of the innate immune response for disease severity in patients with fUTI and the higher response in patients with renal involvement, defined by a positive DMSA scan. This study also detected effects on neutrophil activation and neutrophil migration as well as systemic inflammasome activation and effects on adaptive immunity, broadening the response to include the local and systemic effects of infection on a number of proinflammatory and immunoregulatory cytokines in these patients.

Several of the activated cytokines identified by the proteomic screen fulfilled the criteria of biomarkers of fUTI. The combination of IL-1β, IL-8 and IL-6 showed high AUC values, indicating a high predictive accuracy to identify febrile UTI, which would be of interest to localize infection to the urinary tract when diagnosing a child with fever of unknown origin. The usefulness of these responses as biomarkers of recurrences or renal scarring has not been investigated. Procalcitonin, recognized as a possible biomarker for sepsis and severe inflammation, has been proposed to distinguish acute pyelonephritis from cystitis^35^ and urinary neutrophil gelatinase-associated lipocalin to separate UTI from other febrile infections and to predict acute pyelonephritis^36–38^ and renal scarring.^15,39^ These parameters were not identified as markers of acute disease severity in this study. The results suggest that a loss of immune control and excessive responses affecting both innate and adaptive immunity characterize acute pyelonephritis, not the activation of single cytokines.

This study identifies infection-prone infants with fUTI, who develop a cytokine storm response as a potential target population for novel treatment approaches, aimed at correcting their loss of immune control. Recent evidence supports the concept of treating bacterial infections using targeted acute immunotherapy, rather than by removing the bacteria with antibiotics.^40^ In acute pyelonephritis models, the use of small interfering RNA treatment has shown strong protective effects, through inhibition of the transcription factor IRF-7 and the innate immune response.^31^ Recent studies have further identified the NlpD protein targeting RNA polymerase II and the LON protein targeting the MYC transcription factor, as efficient therapeutic agents in the murine acute pyelonephritis model.^41,42^ In both cases, acute inflammation was inhibited in the mice and bacterial clearance was accelerated with similar efficacy as antibiotics. Similar results were obtained in acute cystitis, where an IL-1 receptor antagonist, targeting overactive IL-1β processing machinery, has shown a broad protective effect in the murine acute cystitis model.^12,40,43^ Protective effects were recently clinically validated in a controlled Phase II study, where IL-1RA treatment showed similar efficacy as antibiotics (I. Ambite, A. Pilatz, M. Buch-Heberling, et al, unpublished data).

## ACKNOWLEDGMENTS

*The authors thank the clinical study teams who participated in this study.*

## Supplementary Material
