# Supplementary material for: Analysis of the Innate Immune Response to Febrile UTI in Infants: Evidence of an Acute Cytokine Storm
Source: Pediatr Infect Dis J. 2025 Aug 26;44(12):1188–200. doi: 10.1097/INF.0000000000004914 (PMC12594124; doi:10.1097/INF.0000000000004914)
Supplement: Supplementary file 1 [file inf-44-1188-s001.pdf]

A Demographics and clinical characteristics

	**Febrile UTI (n=55)**
Mean age (months)	
Total	4.2
Male	2.9
Female	5.1
Gender no (%)	
Male	23 (41.8)
Female	32 (58.2)
Fever at inclusion	
Max temperature	39.3
CRP at inclusion	89.2
Method of urine collection	
Clean catch	48
SPA	4
Catheter	3
Urine nitrite no (%)	
Positive	23 (41.8)
Negative	32 (58.2)
Urine Leukocyte esterase	
Negative/trace	3
Positive>1+	52

B Imaging and bacterial cultures

	**Febrile UTI**	**Febrile UTI with *E.coli***	**Febrile UTI with non *E.coli***
DMSA			
1st DMSA	n=55	n=50	n=5
+	33 (60%)	29 (58%)	1 (20%)
-	22 (40%)	21 (42%)	4 (80%)
2nd DMSA	n= 22	n=17	n=5
Renal scarring	7 (32%)	2 (11.8%)	5 (100%)
Resolved APN	15 (68%)	15 (88.2%)	0 (0%)
VCUG finding	n=27	n=22	n=5
No VUR	19 (70.4%)	19 (86.4%)	0 (0%)
Non dilated VUR	2 (7.4%)	1 (4.5%)	1 (20%)
Dilated VUR	6 (22.2%)	2 (9.1%)	4 (80%)

Supplemental Digital Content 1.

Demographics and clinical characteristics of fUTI patients in this study. (A) Table shows age, gender distribution, clinical information, methods for urine collection, urine nitrate and urine leukocyte numbers. (B) Table shows imaging and bacterial findings in infant fUTI patients, based on DMSA scanning and voiding cystourethrogram (VCUG) finding.

A Group wise analysis of median values in acute versus follow up samples (n=29)

	Median		
	Acute	Follow up	*P* value
IL-1β	161.74	2.6	<0.0001
IL-1α	20.14	5.32	<0.0001
IL-1Ra	3715.89	1130.42	0.001
IL-33	190.43	88.99	<0.0001
IL-8	616.78	27.82	<0.0001
IP-10	43.59	2.76	<0.0001
MCP-1	670.78	117.22	<0.0001
MIP1-α	32.74	11.84	0.0005
MIP1-β	258.52	82.81	<0.0001
GM-CSF	94.73	11.43	0.0005
IL-6	55.75	8.12	<0.0001
IL-17	4.45	0.83	<0.0001
TNF-α	2.17	0.58	0.01
IFN-γ	100.97	16.09	<0.0001
IFN-α2	0.76	0.86	0.24
CD40	5983.62	314	<0.0001
IL-2	18.72	2.95	<0.0001
Granzyme B	107.31	14.94	0.009
IL-10	58.57	18.79	<0.0001
IL-15	13.51	3.56	0.0015
PD-L1	1148.03	362.8	0.0002
IL-13	6.21	4.94	0.43
IL-12p70	8.46	1.88	0.39
IL-4	0.08	0.084	0.22

B Group wise analysis of median values of urine samples at enrolment, Group1(n=29) versus Group 2 (n=26)

	Median		
	Group 1	Group 2	*P* value
CD40	5983.62	1666.19	0.02
IL-1Ra	3715.89	5022.14	ns
PD-L1	1148.03	757.87	ns
MCP-1	670.78	1087.7	ns
IL-8	616.78	1416.33	ns
MIP1-β	258.52	363.9	ns
IL-33	190.43	117.08	0.01
IL-1β	161.74	131.83	ns
Granzyme B	107.31	65.91	0.04
IFN-γ	100.97	55.93	0.02
GM-CSF	94.73	98.4	ns
IP-10	43.59	88.7	ns
IL-10	58.57	84.13	ns
IL-6	55.75	66.38	ns
MIP1-α	32.74	39.5	ns
IL-1α	20.14	34.34	ns
IL-2	18.72	19.40	ns
IL-15	13.51	14.46	ns
IL-12p70	8.46	10.50	ns
IL-13	6.21	23.46	0.01
IL-17	4.45	8.64	ns
TNF-α	2.17	7.19	ns
IFN-α2	0.76	2.04	ns

Supplemental Digital Content 2.

(A) Cytokine concentrations in urine, comparing the group used for pairwise analysis (n=29). *P*-values are shown for the immune response to infection (fUTI) compared to follow up samples (Wilcoxon rank test). (B) Median concentrations of urine cytokines in the two study groups, at enrolment. Group1 are patients with follow up samples (n=29) and Group 2 are patients without follow up samples (n=26). *P* values compare the urine protein responses during fUTI between Group 1 and Group 2 (Mann Whitney test).

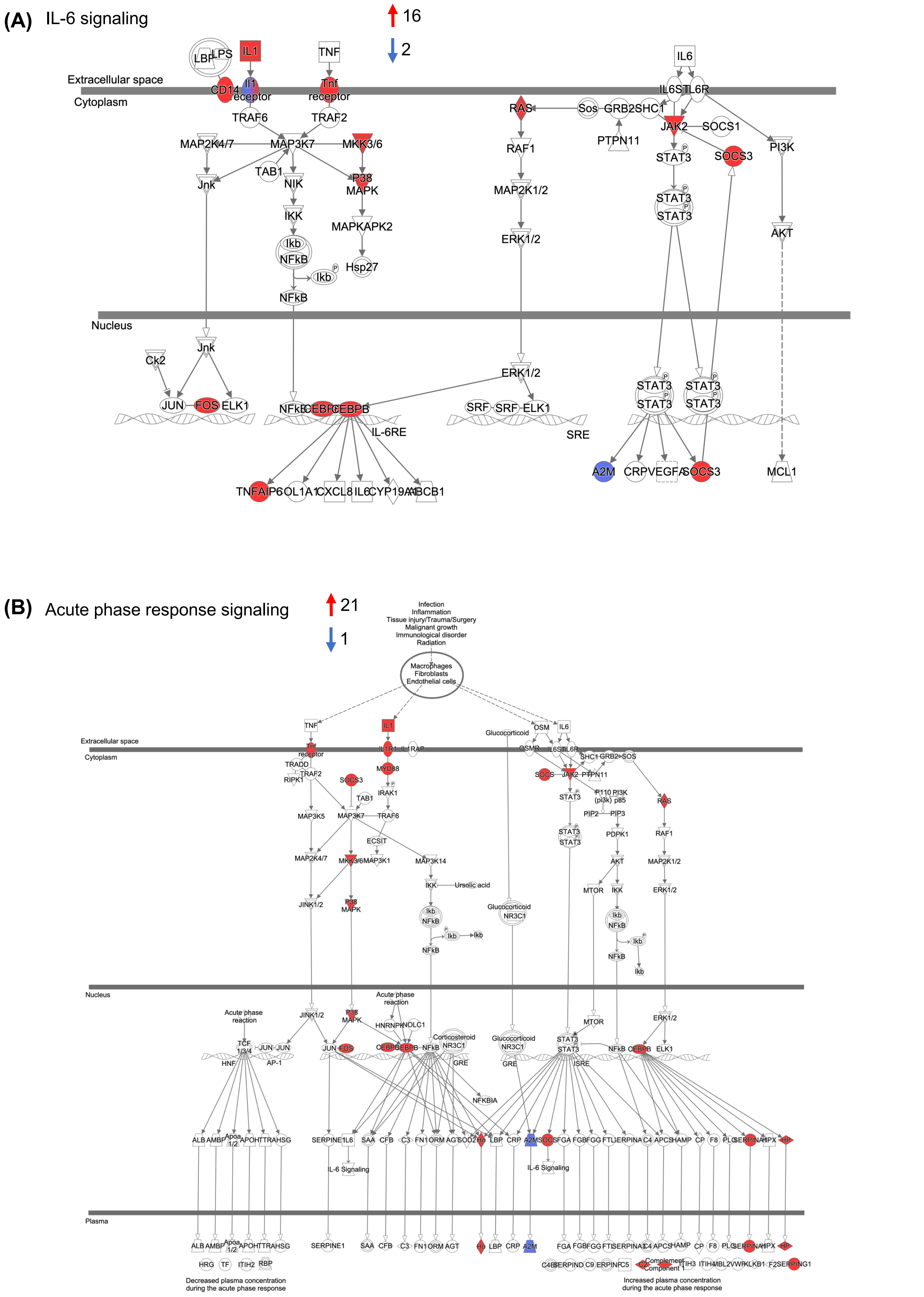

(A) IL-6 signaling
16
2
Extracellular space
Cytoplasm
Nucleus
LBP
LPS
IL1
TNF
IL6
CD14
Il1 receptor
Tnf receptor
IL6ST
IL6R
TRAF6
TRAF2
RAS
Sos
GRB2
SHC1
JAK2
SOCS1
PTPN11
MAP2K4/7
MAP3K7
MKK3/6
RAF1
STAT3
SOCS3
PI3K
TAB1
Jnk
NIK
P38 MAPK
MAP2K1/2
IKK
MAPKAPK2
ERK1/2
AKT
Ikb
NFkB
Hsp27
Ck2
JUN
FOS
ELK1
CEBPB
SRF
IL-6RE
SRE
TNFAIP6
COL1A1
CXCL8
IL6
CYP19A1
ABCB1
A2M
CRP
VEGFA
MCL1
(B) Acute phase response signaling
21
1
Infection
Inflammation
Tissue injury/Trauma/Surgery
Malignant growth
Immunological disorder
Radiation
Macrophages
Fibroblasts
Endothelial cells
Extracellular space
Cytoplasm
Nucleus
Plasma
TNF
IL1
Glucocorticoid
OSM
IL6
Tnf receptor
IL1R1
IL1RAP
OSMR
IL6ST
IL6R
TRADD
TRAF2
RIPK1
SOCS3
MYD88
SHC1
GRB2
SOS
SOCS
JAK2
PTPN11
IRAK1
TAB1
STAT3
P110 (pi3k)
PI3K p85
RAS
MAP3K5
MAP3K7
TRAF6
PIP2
PIP3
RAF1
ECSIT
PDPK1
MAP2K4/7
MKK3/6
MAP3K1
MAP3K14
AKT
MAP2K1/2
IKK
Ursolic acid
MTOR
ERK1/2
JINK1/2
P38 MAPK
Glucocorticoid NR3C1
Ikb
NFkB
Acute phase reaction
HNRNPK
NOLC1
Corticosteroid NR3C1
TCF 1/3/4
JUN
FOS
CEBPB
ELK1
HNF
AP-1
GRE
ISRE
NFKBIA
ALB
AMBP
Apoa 1/2
APOH
TTR
AHSG
SERPINE1
SAA
CFB
C3
FN1
ORM
AGT
SOD2
Ho
LBP
CRP
A2M
FGA
FGB
FGG
FTL
SERPINA3
C4
APCS
HAMP
CP
F8
PLG
SERPINA1
HPX
HP
IL-6 Signaling
HRG
TF
ITIH2
RBP
C4BP
SERPIND
C9
SERPINF
C5
C2
Complement Component 1
ITIH3
ITIH4
MBL2
VWF
KLKB1
F2
SERPING1
Decreased plasma concentration during the acute phase response
Increased plasma concentration during the acute phase response

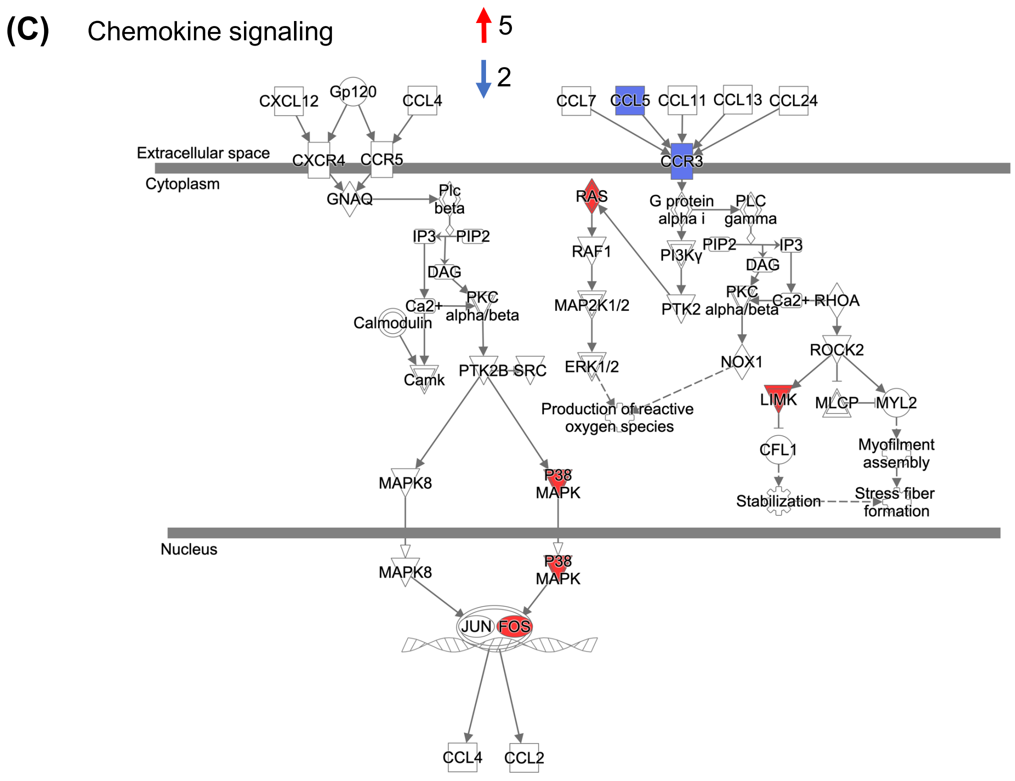
(C) Chemokine signaling
5
2
CXCL12
Gp120
CCL4
CCL7
CCL5
CCL11
CCL13
CCL24
Extracellular space
Cytoplasm
CXCR4
CCR5
CCR3
GNAQ
Plc beta
IP3
PIP2
DAG
Ca2+
PKC alpha/beta
Calmodulin
Camk
PTK2B
SRC
RAS
RAF1
MAP2K1/2
ERK1/2
G protein alpha i
PLC gamma
PI3Kγ
PIP2
IP3
DAG
PKC
PTK2 alpha/beta
Ca2+
RHOA
ROCK2
NOX1
Production of reactive oxygen species
LIMK
MLCP
MYL2
CFL1
Myofilment assembly
Stabilization
Stress fiber formation
MAPK8
P38 MAPK
Nucleus
MAPK8
P38 MAPK
JUN
FOS
CCL4
CCL2

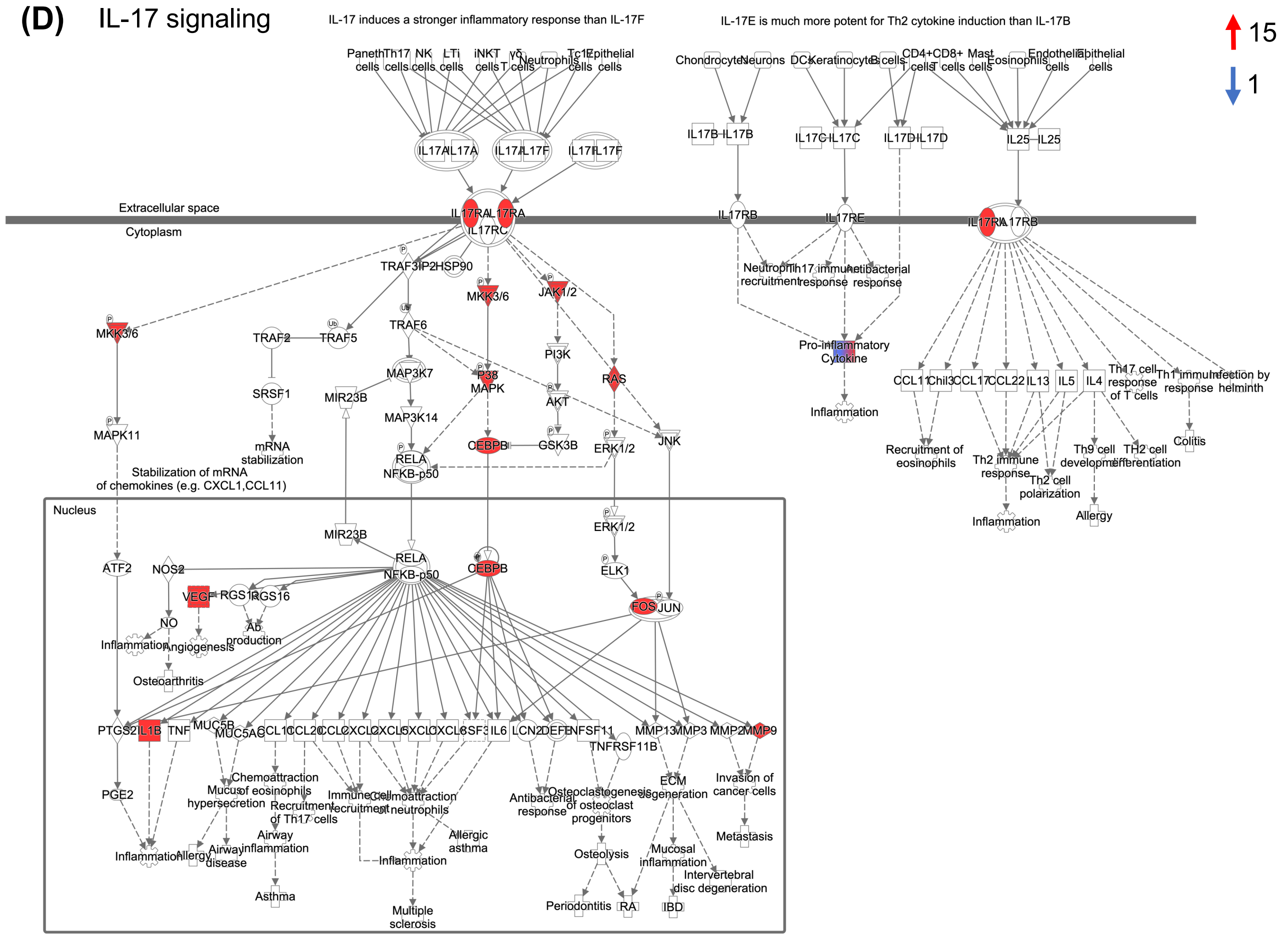
(D) IL-17 signaling
IL-17 induces a stronger inflammatory response than IL-17F
IL-17E is much more potent for Th2 cytokine induction than IL-17B
15
1
Extracellular space
Cytoplasm
Nucleus
IL17RA
IL17RC
IL17RB
IL17RE
TRAF3IP2
HSP90
MKK3/6
JAK1/2
TRAF2
TRAF5
TRAF6
SRSF1
MIR23B
MAP3K7
MAP3K14
P38 MAPK
PI3K
AKT
GSK3B
RAS
CEBPB
ERK1/2
JNK
RELA NFKB-p50
MAPK11
mRNA stabilization
Stabilization of mRNA of chemokines (e.g. CXCL1,CCL11)
Pro-inflammatory Cytokine
Inflammation
ATF2
NOS2
VEGF
RGS16
ELK1
FOS
JUN
PTGS2
IL1B
TNF
MUC5B
MUC5AC
IL6
TNFRSF11B
MMP13
MMP3
MMP2
MMP9
PGE2
Inflammation
Allergy
Asthma
Osteoarthritis
Angiogenesis
Chemoattraction of eosinophils
Mucus hypersecretion
Airway inflammation
Allergic asthma
Multiple sclerosis
Osteolysis
Periodontitis
RA
IBD
ECM degeneration
Mucosal inflammation
Intervertebral disc degeneration
Invasion of cancer cells
Metastasis
Recruitment of eosinophils
Th2 immune response
Th2 cell polarization
Colitis
Allergy

(E) NF-κB signaling

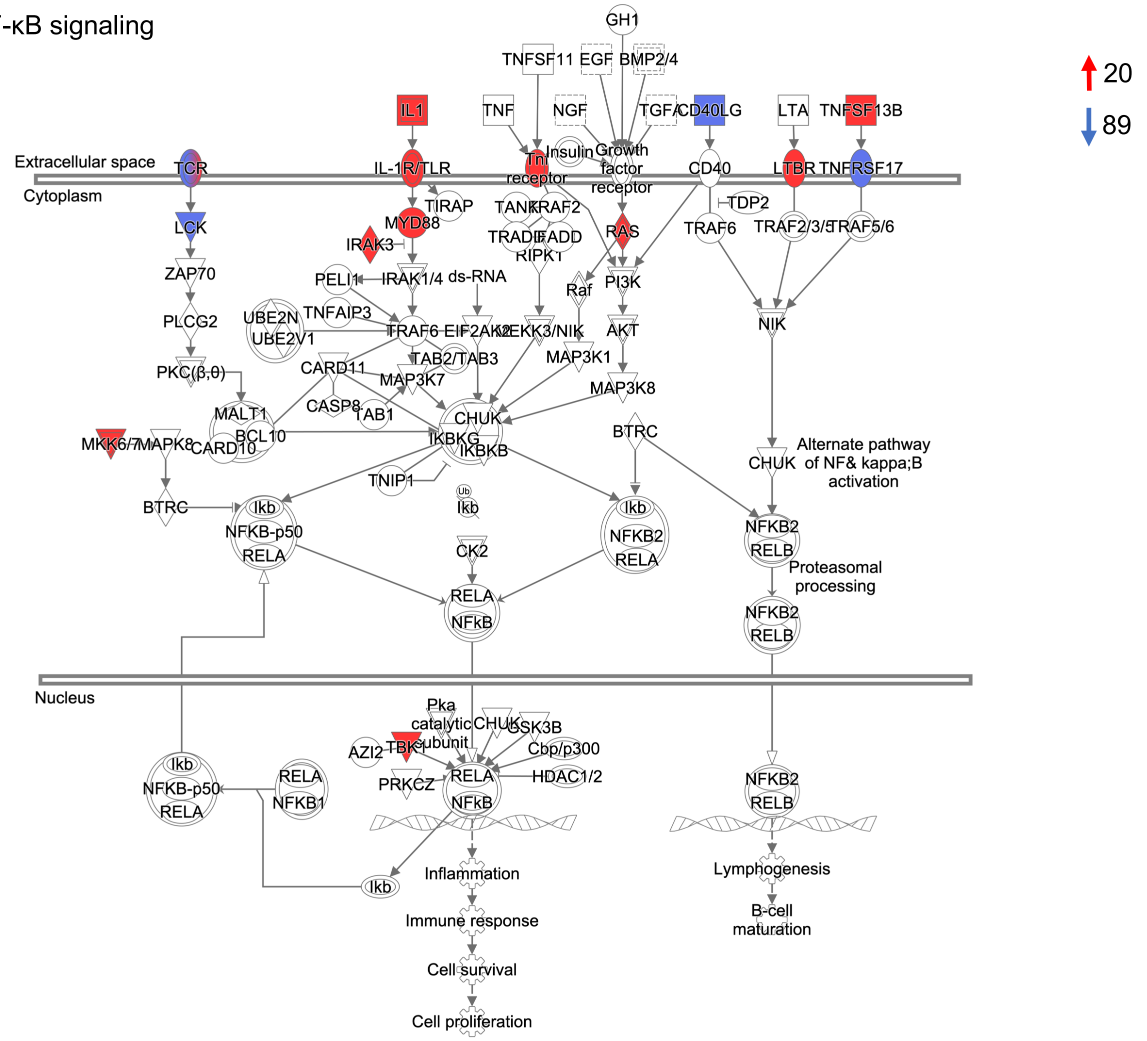

Supplemental Digital Content 3.

(A, B) Upregulation of majority of immune genes was observed in IL-6 signaling (16 upregulated genes, 2 downregulated) and Acute phase response signaling (21 upregulated genes, 1 downregulated) in fUTI patients in the time of infection compared to follow up. (C, D) Chemokine signaling (5 upregulated genes, 2 downregulated) and IL-17 signaling (15 upregulated genes, 1 downregulated). Upregulation of majority of immune genes in fUTI patients was observed in the time of infection compared to follow up. (E) Upregulation of 20 and downregulation of 89 immune genes was observed for NF-κB signaling in fUTI patients in the time of infection compared to follow up.

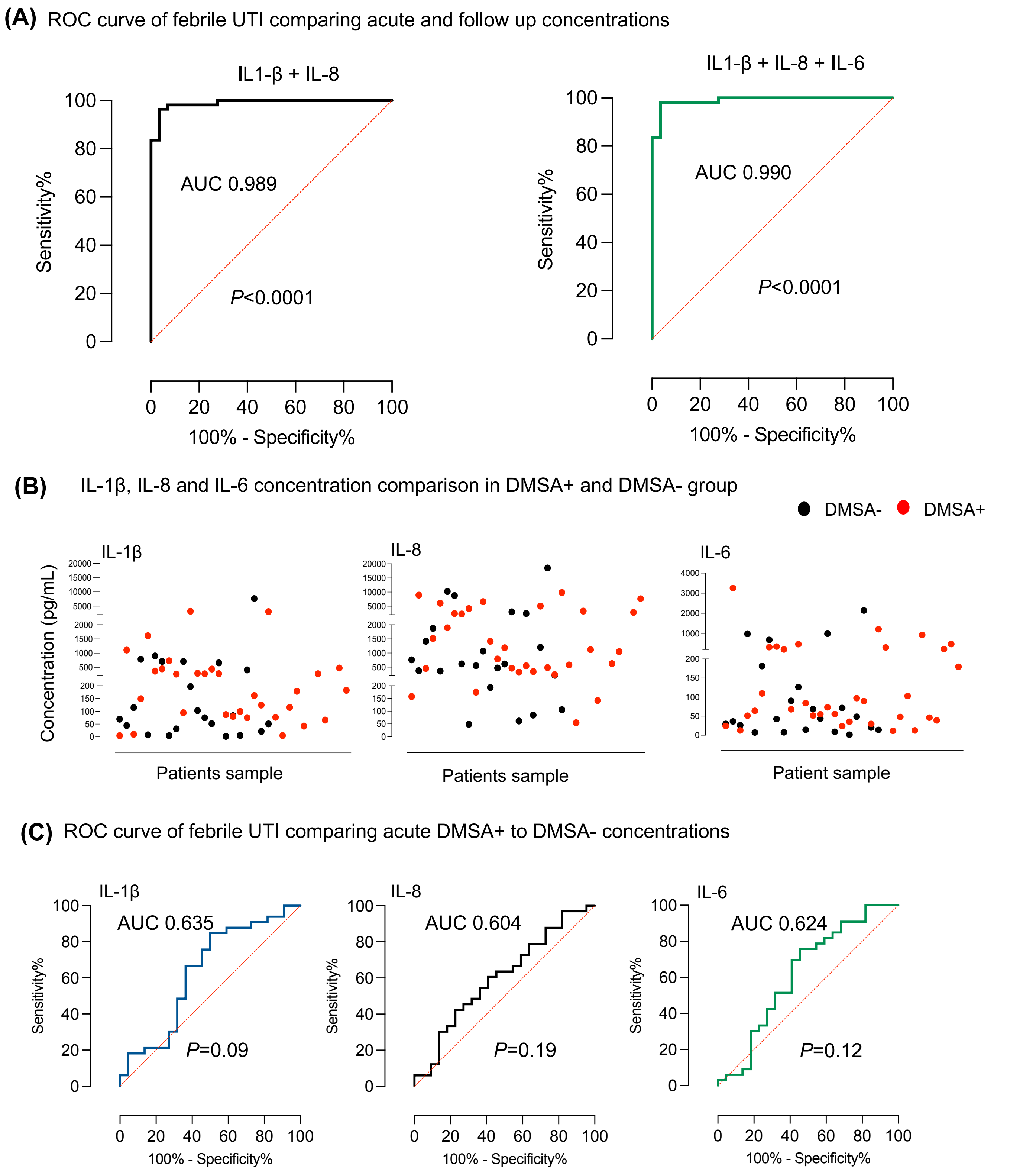

Supplemental Digital Content 4.

(A) ROC curve of febrile UTI comparing acute and follow up concentrations. With an AUC of > 0.989 for IL-1β and IL-8, and an AUC of > 0.990 for the combination of IL-1β, IL-8 and IL-6, the ROC curves indicated that these proteins have a high predictive accuracy to discriminate the febrile UTI from the follow up groups with potential as biomarkers. (B) Urine concentration comparison of IL-1β, IL-8 and IL-6 at the enrolvmet in DMSA+ and DMSA- group. No significant difference was observed in protein concentration for IL-1β, IL-8 and IL-6. (C) ROC curve of febrile UTI comparing acute DMSA+ to DMSA- concentrations . With an AUC of 0.6 for IL-1β ,IL-8, and IL-6, with $P > 0.05$ the ROC curves for IL-1β, IL-8 and IL-6. These proteins did not have a high predictive accuracy to discriminate the DMSA+ from the DMSA- groups as potential biomarkers.
